# GeneProf data: a resource of curated, integrated and reusable high-throughput genomics experiments

**DOI:** 10.1093/nar/gkt966

**Published:** 2013-10-29

**Authors:** Florian Halbritter, Anastasia I. Kousa, Simon R. Tomlinson

**Affiliations:** Institute for Stem Cell Research, Centre for Regenerative Medicine, School of Biological Sciences, University of Edinburgh, SCRM Building, 5 Little France Drive, Edinburgh EH16 4UU, UK

## Abstract

GeneProf Data (http://www.geneprof.org) is an open web resource for analysed functional genomics experiments. We have built up a large collection of completely processed RNA-seq and ChIP-seq studies by carefully and transparently reanalysing and annotating high-profile public data sets. GeneProf makes these data instantly accessible in an easily interpretable, searchable and reusable manner and thus opens up the path to the advantages and insights gained from genome-scale experiments to a broader scientific audience. Moreover, GeneProf supports programmatic access to these data via web services to further facilitate the reuse of experimental data across tools and laboratories.

## INTRODUCTION

High-throughput profiling technologies such as microarrays and, more recently, next-generation sequencing (NGS) have become invaluable tools for biomedical research. Their popularity is reflected by the ever-increasing growth of the associated primary data archives, most prominently ArrayExpress (1), GEO (2) and the databases of the International Nucleotide Sequence Database Collaboration (3–5). Simply archiving the data, however, is not sufficient to make it immediately accessible to the scientific community. The sheer amount and complexity of the data make it challenging to process, analyse and interpret. We and others have therefore developed user-friendly software facilitating streamlined analysis of large quantities of high-throughput data (6–8), but nevertheless much time is spent analysing the same data sets in different laboratories.

To reduce further replication of efforts and to make state-of-the-art insights from genome-wide experiments instantly interpretable to scientists, we have started building up a database of high-profile functional genomics data sets as a resource for biomedical research. To this end, we have used the web-based GeneProf data analysis system (6) to carefully reanalyse and curate a large number of public data sets and to bring them all together under one common framework. We have paid special attention to make this resource useful for experimental and computational biologists alike: the data can either be browsed, searched and visualized via the website or retrieved programmatically using a collection of web services. Importantly, thanks to its integration into the broader GeneProf data analysis suite, each result in the database is connected with the full analysis workflow that was used to generate it and all data can immediately be reused in new projects and integrated with the users' own data to enrich their results.

## DATABASE CONTENT

### Data sources, types and extent

**Figure 1.**
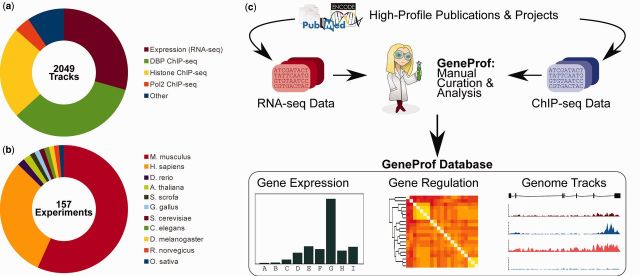
(**a** and **b**) The GeneProf database currently hosts data from 157 experiments in a variety of organisms (a) and 2049 genome browser tracks (b), mostly from various types of ChIP-seq experiments. DBP = DBP. Data collected on 30 July 2013. (**c**) Schematic overview of data collection, analysis and current content of the database.

In its current form, the GeneProf database hosts gene expression data from RNA-seq experiments as well as regulatory data from ChIP-seq, such as transcription factor binding sites and their putative targets or genes with enriched histone marks (Figure 1a). GeneProf covers data from a range of different organisms, but the vast majority of data comes from human and mouse (Figure 1b). GeneProf is actively being used by our group and others and the database is constantly growing as new data is being made available.

At the time of writing, GeneProf's collection of high-throughput data comprises >76 billion sequence reads from 2617 sequencing experiments, or a total of ∼7 TB of public data (30 July 2013, Supplementary Table S1). The data have been manually selected by the authors from 139 publications and a number of large-scale projects [e.g. ENCODE (9,10) or the Epigenomics Roadmap (11)] with the aim to cover a wide range of diverse data sets of interest to the community. Further data sets are constantly being added by our team, and users may even contribute their own data.

All data in the system has been reanalysed, curated and annotated by the authors and other contributors (Figure 1c). Where possible we have imported available annotations from the source databases, but all experiments have additionally been curated by hand to complete missing annotations and to make all annotations more consistent across experiments. Analysis results and data sets are further supplemented with plots and visualizations to support data interpretation.

### Transparent pipelines

All experiments in the GeneProf Data collection have been completely reanalysed by the authors. In doing so, we have not simply attempted to recapitulate the analysis as presented in the original research publications, but we rather followed a consistent analysis procedure to improve the comparability between independent experiments. The exact workflows used for the analysis vary slightly between projects, as each workflow has been carefully customized for the particular data sets in question. This is essential owing to the wide variety of applications of NGS technology and the resulting differences between experiments. However, most experimental workflows conform closely to the standard analysis suggested by GeneProf's workflow generation wizards (6) and are then fine-tuned with individual parameter adjustments. As such, we have made use of tried-and-tested popular algorithms such as TopHat (12) and DEseq (13) for RNA-seq analysis and Bowtie (14) and MACS (15) for ChIP-seq data.

**Figure 2.**
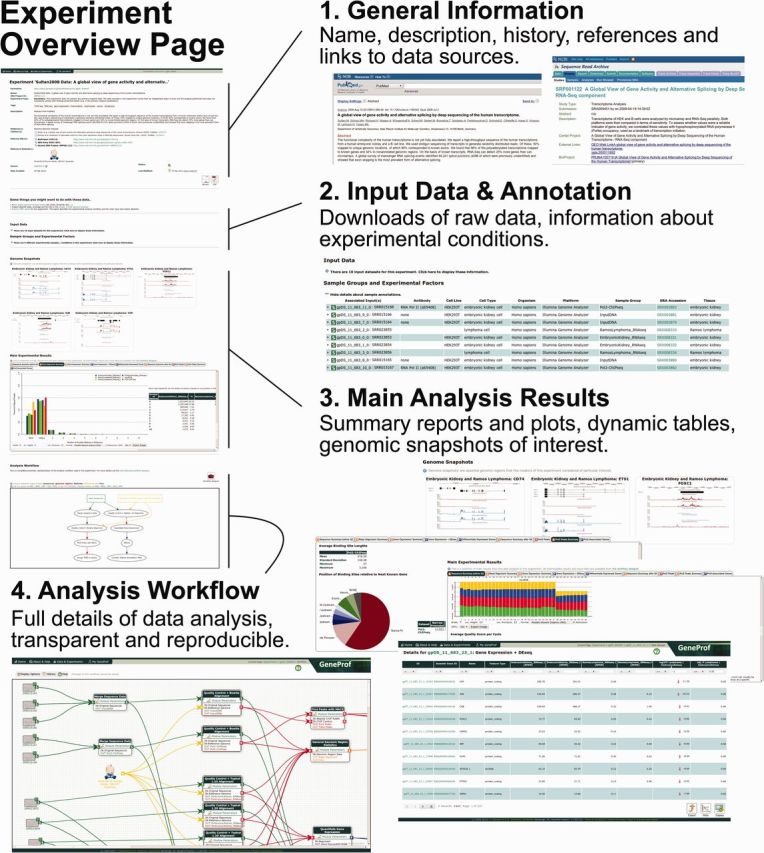
Collage of screenshots from an experiment overview page. Experiments organize related data sets together and combine them with annotations, an analysis workflow and analysis outputs. The full page depicted in the figure is available at http://www.geneprof.org/show?id=gpXP_000683.

Unfortunately, issues with the reproducibility of bioinformatic analysis of high-throughput data sets are pervasive (16). To address part of these problems, the GeneProf database therefore couples all results with the corresponding analysis workflows and quality control measures, so it is possible to recapitulate each step of the analysis and trace the origin of every single piece of data in the system (Figure 2). This may help to increase the long-term impact of research projects (17).

Data sets in the GeneProf database are organized in terms of ‘experiments’, where each experiment typically corresponds to a publication or another logically linked set of sequencing experiments. With each experimental workflow, the database stores not only the analysis results, but also the raw input data (including references to publications and links to the original data sources) and all intermediate outputs; so neither materials nor methods will ever go missing.

## INTERFACES

### Graphical web application

The primary user interface to the GeneProf database is via an open web application that provides access to all central components of the underlying database (http://www.geneprof.org):

#### Experiment overview pages

To make it straightforward for researchers to find all information linked to a particular experiment, we provide a one-stop summary page for each experiment in the database (Figure 2). Along with a name, description and keywords, this page records the references to associated articles and external websites, the owner of the experiment (the person who uploaded the data and carried out the analysis) and to the analysis history of the experiment (a complete log of each analysis step carried out in the past). The page further contains a listing of all input data sets along with sample annotations for each input data set, detailing experimental factors and other parameters, for example, organism, tissue and cell type of origin of each sample, where applicable. The next section of the page lists selected main analysis results of the experimental workflow, as chosen by the creator of the project. Typically, these will include summary reports covering the raw sequence data, alignment, gene expression or an overview of putative binding sites (‘peaks’) discovered, as well as chosen output data sets like tables of expressed genes or lists of binding sites. The system provides a range of automatically generated visualizations to support data interpretation. Each of these result data sets come with additional links to the respective data set pages, detailed below. The last item on the page is a simplified illustration of the analysis workflow giving users an overview of the crucial steps of the analysis process. Full details are available via a link to the detailed analysis workflow, which enables users to drill down into the minutiae of the analysis, including the values for every parameter used.

#### Data set pages

Each experiment consists of many different input, intermediate and output data sets. Each of these data sets comes with a linked data set page. Most records in the GeneProf database are of a tabular nature and can be browsed, sorted and filtered dynamically via the website (Supplementary Figure S1). For example, a table may be filtered by *P*-value to look for differentially expressed genes in an expression data set. Alternatively, all data sets can be exported in a variety of popular file formats (e.g. FASTA/Q, BED, WIG, CSV, XLS, XML or Rdata), so users can transfer the data into their favourite applications. Additionally, integrated plotting tools make it possible to create publication-quality scatter plots, histograms, heatmaps, pie charts, box plots and Venn diagrams directly from within the application.

#### Gene-centric summary reports

**Figure 3.**
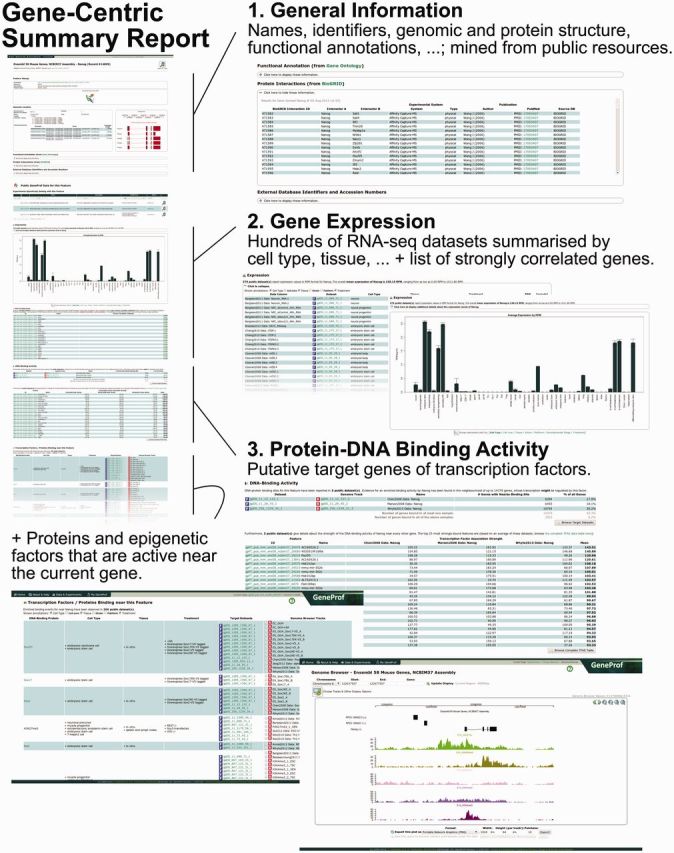
Collage of screenshots from a gene report page. These pages provide one-stop access to all the data GeneProf stores about a particular gene, covering its expression in different conditions and putative regulatory signals acting on it. The page depicted in the figure is available at http://www.geneprof.org/show?id=gpFT_pub_mm_ens58_ncbim37_14899.

Many scientists are interested in obtaining information concerning a particular gene, but lack the time and expertise to exploit the wealth of knowledge buried in high-throughput data sets. GeneProf summarizes all the functional data contained in its databases on a gene-centric level, which makes it easy to instantly benefit from the results of genomics experiments (Figure 3). Each gene summary page first repeats general information about the gene, such as names and identifiers, genomic location, protein structure, known protein interactions and functional annotation. This information is mined from the Ensembl (18), PDB (19), BioGRID (20) and Gene Ontology (21) databases. The remainder of the page displays GeneProf-specific data, starting with gene expression (based on RNA-seq experiments in the database): A simple bar chart shows the average expression level of the chosen gene across different categories of samples, where the user can choose the categorization criterion, e.g. cell type or tissue. Full details of the expression levels in all individual samples together with links to the source data sets are also available. To facilitate the discovery of related genes, GeneProf also lists other genes with highly correlated expression patterns across all data sets. For transcription factors and other DNA-binding proteins (DBPs) for which ChIP-seq data are available in the system, the page will next list all the data sets reporting genes that might be affected by the binding of the chosen protein. The user may choose to browse the complete list of putative target genes from this point. The last section of the page outlines all DBPs that show evidence of enriched binding in the proximity of the current gene, i.e. those that might be actively involved in regulating the activity of the gene of interest. To examine the genomic landscape in detail, the user can pick a selection of these proteins and view the associated data in the integrated genome browser program (see below).

#### System-wide search

A search page allows users to rapidly locate experiments, data sets and genes of interest (Supplementary Figure S2). The user can either use simple search terms or formulate complex queries powered by an Apache Lucene-based search system (http://lucene.apache.org/). Checkboxes may be used to restrict the search space with a few mouse clicks and many advanced search examples are provided on the page itself.

#### Integrated Genome Browser

Genome browsers are powerful and popular tools to visually explore large-scale genomics data sets. We have built in a simple genome browser directly into GeneProf (Supplementary Figure S3), which is based on GenomeGraphs (22). Using this tool, it is straightforward to quickly visualize genomic data sets in the context of ongoing experiments or other public data. In its current release, the GeneProf database hosts 2049 genome browser tracks (30 July 2013).

If desired, genomic data sets can still be exported in WIG and BED format, so that they can be opened in more powerful external applications, e.g. the UCSC genome browser (23) or IGV (24).

In addition to these main feature pages, GeneProf hosts utility pages for user management, gene identifier conversion and genome information (‘Genome Trivia’; Supplementary Figure S4).

### Web services

As an alternative to the standard graphical access to the GeneProf database, we have implemented a range of web services that enable programmatic access to the data (Supplementary Table S2). As such, the web services allow computational biologists and software developers to use data from outside the GeneProf application itself and to wire the data directly into external analysis pipelines and tools.

GeneProf web services may be broadly subdivided into five categories:

- Search: Lookup of matching database records.
- Metadata: General information about experiments and data sets.
- Gene expression: Gene-centric summaries of all available expression data.
- Gene regulation: Overview of the targets of DBPs.
- Data retrieval: Retrieval of individual data sets in genomic, sequence or text data formats.

We have supplemented the detailed descriptions of the web services offered on the website with a wide range of example applications (http://www.geneprof.org/webapi.jsp). In addition to providing example code in Perl, R, Java and HTML/JavaScript programming languages, we demonstrate the use of the web services with Taverna (25), Galaxy (7) and various genome browser tools (23,24).

## EXAMPLE APPLICATIONS

To illustrate the utility of the GeneProf database, we will outline a few selected example applications in the following section.

### Gathering data about genes of interest

The transcription factor Nanog is a central component of the transcriptional network of embryonic stem cells (ESCs) (26). Using the GeneProf search page (Supplementary Figure S2), the user can easily find the records corresponding to the Nanog gene in all supported organisms by just entering ‘nanog’ into the search box and ticking the boxes for the organisms of interest (example: ‘*Mus musculus*’ and ‘*Homo sapiens*’).

By clicking on the name ‘Nanog’ in the search results, the gene summary page will open. Browsing through the information on the page, a researcher can quickly find out that, for example, Nanog is most highly expressed in ESCs and that its expression is turned on as early in development as the four-cell stage blastomere. Closely correlated genes include the functionally related transcription factor Esrrb (27). The GeneProf database currently contains three ChIP-seq data sets profiling Nanog in mouse ESCs. By clicking the ‘Browse Target Datasets’ button on the gene page and filtering the dynamic table on the next page for genes positively bound in all three data sets, we can identify 2930 putative target genes of Nanog. Returning to the gene page, we find a large number of regulatory elements in the proximity of the Nanog promoter; for instance, the list contains the aforementioned gene Esrrb as well as Nanog itself (with three independent data sets). By ticking the checkboxes for the four data sets concerned and clicking ‘Browse Nanog Locus’, the user can access the genomic neighbourhood of Nanog in the genome browser, from which at least two overlapping regulatory regions for Nanog and Esrrb (upstream of the Nanog promoter) are visually apparent (Supplementary Figure S3).

Further examples can be found in the online manual (http://www.geneprof.org/help_tutorials.jsp#tutorial:ExaminingPublicNext-GenDatausingGeneProf).

### Instant data reuse

Using the GeneProf data analysis suite (6), data sets from the public repository can be instantly integrated into ongoing experiments. This may enrich new projects by adding additional data without significantly increasing the cost and time required.

In our previous work (27), we have found it useful to combine the Tag-seq gene expression data generated as part of our investigations of the downstream targets of Nanog with publicly available ChIP-seq data sets to prioritize the identification of putative direct target genes of this transcription factor. To do so, we have imported the binding peak data sets from two studies in the GeneProf database into our analysis workflow, mapped these peaks to the genes and integrated both sources of data with the expression data. The workflow describing this analysis is available as part of experiment gpXP_000385 (http://www.geneprof.org/show?id=gpXP_000385).

We are using similar data integration methods in our day-to-day work and believe that other scientists will likely benefit from a similar approach.

### Integration into external applications

Bioconductor (28) libraries offer an unparalleled breadth of utilities that are widely used by bioinformaticians. Using GeneProf web services, it is trivial to load, for example, gene expression data for a particular gene, cell type or treatment condition directly into an active R session. The RCurl package provides all the functionality required to establish a connection to these web services, which can export data directly in an R-compatible binary format.

Following on from previous examples, we may wish to further investigate the relation between the transcription factors Nanog and Esrrb. Using GeneProf web services, we can load the fully annotated gene expression values from currently 380 mouse RNA-seq data sets (30 July 2013) into R within seconds, making it possible to calculate their global correlation (Pearson correlation ∼0.84) or to produce a scatter plot in moments (Supplementary Figure S5, Supplementary Methods).

GeneProf's web services open up a plethora of possibilities for integrating GeneProf data with external applications and their use is by no means restricted to R. The online documentation of the web service API (http://www.geneprof.org/webapi.jsp) provides further worked-through examples for using the web services from the Unix command line, from the UCSC (23) or IGV (24) genome browsers, from the Galaxy (7) and Taverna (25) workflow engines, in external web applications via HTML and JavaScript and from within programs written in various programming languages (Perl or Java).

## DOCUMENTATION

The GeneProf website contains an extensive online manual detailing all aspects of the analysis system and the associated databases and providing a range of tutorials for new and returning users (http://www.geneprof.org/help_and_tutorials.jsp, http://www.geneprof.org/webapi.jsp and http://www.geneprof.org/screencasts.jsp; see Supplementary Table S3 for further links). Additionally, we have integrated a simple ticketing system enabling registered users to raise questions and concerns. Users raising queries can generally expect a response within one working day.

## FUTURE DIRECTIONS

GeneProf is being actively maintained and expanded by the authors and the community. We are constantly adding new experimental data sets to the public repository. Furthermore, future improvements will see GeneProf hosting a wider range of data including genome-wide DNA methylation and miRNA data sets. We are also working on improved visualization techniques to enhance exploration and interpretation of genome-scale data sets.

## AVAILABILITY

GeneProf is freely available at http://www.geneprof.org and no login is required to access the public data in the system. Registered users may submit their own data and make it publicly available or share it securely with colleagues and collaborators.

## CONCLUSION

The GeneProf database hosts an ever-increasing wealth of functional genomics data of considerable interest to the biomedical research community. Importantly, GeneProf goes beyond merely archiving high-throughput data, aiming to also process these data into useful formats that help to create novel insight. We are making every effort to keep this database up-to-date and as comprehensive as possible and thanks to GeneProf's unique integration of a database component directly with a data analysis suite, the data in the system is immediately and transparently accessible and reusable by researchers around the globe. GeneProf provides straightforward and quick means to exploit large-scale genomics data sets for both experimental and computational biologists and we aim to further improve its utility by adding additional functionality and data in future releases.

## SUPPLEMENTARY DATA

Supplementary Data are available at NAR Online.

## FUNDING

This work was supported by the Medical Research Council [G0901533]; initial development supported by a Medical Research Council studentship (to F.H.); EU FP7 project EuroSyStem. Funding for open access charge: Medical Research Council (UK) to the Centre of Regenerative Medicine.

*Conflict of interest statement*. None declared.
